# Sex differences in cardiovascular complications and mortality in hospital patients with covid-19: registry based observational study

**DOI:** 10.1136/bmjmed-2022-000245

**Published:** 2023-02-14

**Authors:** Carinna Hockham, Marijke Linschoten, Folkert W Asselbergs, Chahinda Ghossein, Mark Woodward, Sanne A E Peters, the CAPACITY-COVID Collaborative Consortium, AK Al-Ali

**Affiliations:** 1 The George Institute for Global Health, Imperial College London, London, UK; 2 The George Institute for Global Health, University of New South Wales, Sydney, New South Wales, Australia; 3 Department of Cardiology, Division Heart and Lungs, University Medical Centre Utrecht, Utrecht, Netherlands; 4 Department of Cardiology, Amsterdam University Medical Centers, University of Amsterdam, Amsterdam, Netherlands; 5 Health Data Research UK and Institute of Health Informatics, University College London, London, UK; 6 Department of Cardiology, Maastricht University Medical Centre, Maastricht, Netherlands; 7 Department of Obstetrics and Gynecology, School for Oncology and Developmental Biology (GROW), Maastricht University Medical Center, Maastricht, Netherlands; 8 Cardiovascular Research Institute Maastricht (CARIM), Maastricht University, Maastricht, Netherlands; 9 Julius Centre for Health Sciences and Primary Care, University Medical Centre Utrecht, Utrecht, Netherlands

**Keywords:** COVID-19, epidemiology, heart failure, cardiology

## Abstract

**Objective:**

To assess whether the risk of cardiovascular complications of covid-19 differ between the sexes and to determine whether any sex differences in risk are reduced in individuals with pre-existing cardiovascular disease.

**Design:**

Registry based observational study.

**Setting:**

74 hospitals across 13 countries (eight European) participating in CAPACITY-COVID (Cardiac complicAtions in Patients With SARS Corona vIrus 2 regisTrY), from March 2020 to May 2021

**Participants:**

All adults (aged ≥18 years), predominantly European, admitted to hospital with highly suspected covid-19 disease or covid-19 disease confirmed by positive laboratory test results (n=11 167 patients).

**Main outcome measures:**

Any cardiovascular complication during admission to hospital. Secondary outcomes were in-hospital mortality and individual cardiovascular complications with ≥20 events for each sex. Logistic regression was used to examine sex differences in the risk of cardiovascular outcomes, overall and grouped by pre-existing cardiovascular disease.

**Results:**

Of 11 167 adults (median age 68 years, 40% female participants) included, 3423 (36% of whom were female participants) had pre-existing cardiovascular disease. In both sexes, the most common cardiovascular complications were supraventricular tachycardias (4% of female participants, 6% of male participants), pulmonary embolism (3% and 5%), and heart failure (decompensated or de novo) (2% in both sexes). After adjusting for age, ethnic group, pre-existing cardiovascular disease, and risk factors for cardiovascular disease, female individuals were less likely than male individuals to have a cardiovascular complication (odds ratio 0.72, 95% confidence interval 0.64 to 0.80) or die (0.65, 0.59 to 0.72). Differences between the sexes were not modified by pre-existing cardiovascular disease; for the primary outcome, the female-to-male ratio of the odds ratio in those without, compared with those with, pre-existing cardiovascular disease was 0.84 (0.67 to 1.07).

**Conclusions:**

In patients admitted to hospital for covid-19, female participants were less likely than male participants to have a cardiovascular complication. The differences between the sexes could not be attributed to the lower prevalence of pre-existing cardiovascular disease in female individuals. The reasons for this advantage in female individuals requires further research.

**WHAT IS ALREADY KNOWN ON THIS TOPIC**

- Female individuals with covid-19 disease have a lower risk of respiratory failure, admission to hospital, or death than male individuals
- Whether these sex differences in covid-19 disease extend to cardiovascular complications is unclear, or the extent to which differences are explained by the lower prevalence of pre-existing cardiovascular disease in female individuals

**WHAT THIS STUDY ADDS**

- Female participants admitted to hospital for covid-19 had a lower risk of arrhythmia, cardiac ischaemia, pulmonary embolism, or death than male participants while in hospital
- These differences between the sexes persisted in patients with pre-existing cardiovascular disease and might not be explained by the lower prevalence of cardiovascular disease in female participants
- No difference between the sexes was seen for the risk of heart failure or stroke

**HOW THIS STUDY MIGHT AFFECT RESEARCH, PRACTICE, OR POLICY**

- Further research is needed to better understand the male disadvantage in covid-19, specifically whether the pathophysiological mechanisms of covid-19 itself affect female and male individuals differentially

## Introduction

Covid-19 disease typically manifests as a respiratory illness, but cardiovascular complications have been reported in patients with covid-19. These complications include arrhythmia,^1^ heart failure,^2^ and thromboembolic events.^3^ Risk estimates vary between cardiovascular subtypes, treatment settings, and patient groups, but are generally increased in those admitted to hospital and requiring intensive care.^2 4^

The pre-existence of cardiovascular disease in patients with covid-19 is associated with worse outcomes,^5 6^ although differences between subtypes of cardiovascular disease exist.^7^ In an early study of 44 672 patients with covid-19, the case fatality rate in those with pre-existing cardiovascular disease was 10.5%, compared with 2.3% in the overall cohort.^8^ More recently, a meta-analysis of 51 studies comprising 48 317 patients found that the odds of severe covid-19 (defined as the presence of respiratory distress or the need for intensive care) or death were consistently higher in patients with cardiovascular disease than in those with no previous disease across different age groups.^5^

Sex differences exist in the risk of admission to hospital and death from covid-19, with female individuals reportedly having better outcomes, on average, than male individuals.^9^ At the population level, this difference might in part be explained by the lower prevalence of cardiovascular disease in female individuals. Sex differences in the pathophysiology, progression, and recurrence of cardiovascular disease also exist, however, independent of covid-19.^10–13^ Sex and cardiovascular disease possibly interact to increase or reduce the differences between the sexes in the risk of severe manifestations of covid-19.^14^ Few studies have examined this interaction so far, however, potentially masking clinically important details in our understanding of how female and male individuals are differentially affected by covid-19. For example, in an Italian study of 1683 patients (33% female) admitted to hospital for covid-19 between 1 March and 20 April 2w020, female sex was found to be associated with lower mortality than male sex only in patients with mild coronary calcification (volume <100 mm^3^, a biomarker of the risk of cardiovascular disease^15^) at admission, whereas in patients with moderate-to-severe calcification, no difference between the sexes was found.^16^

Also, most research into sex differences in covid-19 has focused on the risk of admission to hospital or the intensive care unit, respiratory distress, and death.^9^ Less studied is whether cardiovascular complications from covid-19 differ between the sexes, both in terms of their risk and complication profile. Based on data from the Cardiac complicAtions in Patients With SARS Corona vIrus 2 regisTrY (CAPACITY-COVID), we assessed the risk of a range of cardiovascular complications in covid-19 in female and male individuals, and investigated whether sex differences in these risks were modified by pre-existing cardiovascular disease.

## Methods

### Study setting

CAPACITY-COVID is a multinational registry of individuals with highly suspected covid-19 disease or covid-19 disease confirmed by a positive laboratory test result, who were admitted to hospital for covid-19. Details of the registry have been previously described.^7^ Briefly, CAPACITY-COVID involves a standardised data collection tool that extends the ISARIC-WHO (International Severe Acute Respiratory and emerging Infection Consortium-World Health Organization) covid-19 case report form^17^ with about 400 more variables, to collect information on the role of cardiovascular disease in patients with covid-19 (www.capacity-covid.eu). This extra information relates to the patient’s cardiovascular risk profile and pre-existing cardiovascular disease status, use of drug treatments, and cardiovascular outcomes during admission to hospital. Seventy four hospitals in 13 countries (eight European) participate in the registry. Fifty six of these hospitals use a non-selective approach for patient inclusion, where every patient admitted with covid-19 is recruited or a random sample of patients admitted to hospital is included in the registry. The remaining 18 sites only include patients with a history of cardiovascular disease or risk factors for cardiovascular disease, or where a cardiologist was consulted during admission. Patients are followed from hospital admission to discharge, with information held in their electronic health records generated during routine care. Access to the full database was obtained under a data transfer agreement between the University Medical Centre Utrecht and Imperial College of Science, Technology, and Medicine.

In this study, we included all adult patients (≥18 years) with confirmed covid-19 who were admitted to a participating hospital between March 2020 and May 2021 and whose sex at birth and date of admission were available in the registry. Patient-reported gender was not captured in the registry. During this period, the dominant SARS-CoV-2 lineages in Europe were D614G followed by B.1.1.7 (alpha).^18–21^ Covid-19 vaccinations were available from December 2020, with 60% and 40% of the population receiving their first dose by June 2021 in the UK and the Netherlands, respectively.^20 22 23^

### Outcomes

Our primary outcome was any cardiovascular complication during the patient’s hospital admission for covid-19. Complications collected in the registry included myocarditis, pericarditis, endocarditis, arrhythmia (including conduction disorders), cardiac ischaemia, heart failure, stroke, and pulmonary embolism. Myocarditis, pericarditis, endocarditis, and acute coronary syndrome were defined according to the diagnostic criteria of the corresponding European Society of Cardiology guidelines (online supplemental table 1). For arrhythmias, definitions were based on the American College of Cardiology-American Heart Association-Heart Rhythm Society 2006 data standards (online supplemental table 1).

10.1136/bmjmed-2022-000245.supp1 Supplementary data

Secondary outcomes were in-hospital mortality and subtypes of cardiovascular complications with ≥20 events in both sexes. For arrhythmias, we examined all arrhythmias together as well as supraventricular tachycardia on its own. For heart failure, we examined any occurrence of heart failure (decompensated and de novo), and de novo heart failure on its own.

### Definition of pre-existing cardiovascular disease

Pre-existing cardiovascular disease was defined as any recorded history of one or more of the following diagnoses before covid-19: arrhythmia or conduction disorder, heart failure, coronary artery disease, valvular disease, and congenital heart disease. Guideline based definitions of the diagnoses were provided in the case report form, with assessment based on information held within the patients’ electronic health records.

### Statistical methods

Baseline characteristics, including medical history, symptoms, measurements of vital signs, and laboratory measurements at admission, and length of stay in hospital and in the intensive care unit, were summarised with standard measures, and grouped by sex and pre-existing cardiovascular disease status. Missing data for the outcome, exposure, and confounder variables were imputed with multiple imputation with chained equations.^24^ Online supplemental table 2 lists the variables included in the imputation model. Thirty imputed datasets, with 10 iterations each, were generated. Logistic regression was performed on each imputed dataset and the resulting estimates pooled with Rubin’s rules.

We examined the association between sex and each outcome, with four sets of model adjustments: no adjustment; adjusted for age and ethnic group; adjusted for age, ethnic group, history of cardiovascular disease, and use of relevant drug treatment for cardiovascular disease (online supplemental tables 1 and 3); and adjusted for age, ethnic group, history of cardiovascular disease, use of drug treatment for cardiovascular disease, and risk factors for cardiovascular disease (body mass index, diabetes, hypertension, peripheral arterial disease, and dyslipidaemia). To examine whether these associations were modified by pre-existing cardiovascular disease, we included an interaction term between each variable and pre-existing cardiovascular disease obtaining, for each outcome, the female-to-male odds ratio in those with no pre-existing cardiovascular disease and those with pre-existing cardiovascular disease. We divided the odds ratio in the non-cardiovascular disease cohort by the odds ratio in the cardiovascular disease cohort to obtain the ratio of odds ratios.

Analyses were repeated with a complete case analysis and after excluding 794 patients from sites that used selective patient recruitment. Data analysis was performed in R Studio (version 1.4.1717). The online supplemental material provides a list of the statistical packages used.

### Patient and public involvement

Owing to funding constraints, no patients or members of the public were involved in the design, conduct, or reporting of the study. The study results will not be disseminated directly to patients included in the registry because of the de-identified nature of the data. The results will be shared with all participating sites, on social media, and on the CAPACITY-COVID website. The online supplemental material has a lay summary of the study results.

## Results

### Baseline characteristics

Between March 2020 and May 2021, 11 167 patients with confirmed covid-19 were recruited into CAPACITY-COVID (online supplemental figures 1 and 2). Most patients (9568/10 866, 88%) were recruited in the Netherlands or the UK, 74.6% (7457/9995) of patients were white, and 40% (4438/11 167) were female. Median age was 69 years (interquartile interval 55-80) and 67 years (55-77) for female and male individuals, respectively (table 1). The incidence of diabetes (about 26%) was similar in female and male participants (1129/4384 *v* 734/6624), a slightly higher percentage of female participants had hypertension (2032/4345, 47% *v* 2919/6551, 45% in male participants), and a smaller percentage had dyslipidaemia (1155/4168, 28% *v* 2060/6322, 33%). The prevalence of pre-existing cardiovascular disease was lower in female participants (1217/3944, 31% *v* 2206/6153, 36% in male participants), mainly because of their decreased prevalence of coronary artery disease (348/3939, 9% *v* 1068/6148, 17%). Arrhythmia was the most common cardiovascular disease subtype among female participants at admission (531/3939, 14%), whereas for male participants, coronary artery disease predominated. In general, in both sexes, those with pre-existing cardiovascular disease were older and had a higher prevalence of comorbidities (online supplemental table 4).

**Table 1:** Baseline characteristics of CAPACITY-COVID (Cardiac complicAtions in Patients With SARS Corona vIrus 2 regisTrY) participants, by sex

Characteristics	All patients (n=11 167)	Female individuals (n=4438)	Male individuals (n=6729)	P value^*^
**Personal characteristics**				
Mean (SD) age (years)	65.9 (16.2)	66.5 (17.3)	65.5 (15.4)	—
Median (IQI) age (years)	68.0 (55.0-78.0)	69.0 (55.0-80.0)	67.0 (55.0-77.0)	0.01
Age group (years):				—
18-25	133 (1.2)	67 (1.5)	66 (1.0)	
26-35	430 (3.9)	216 (4.9)	214 (3.2)	
36-45	725 (6.5)	293 (6.6)	432 (6.4)	
46-55	1525 (13.7)	553 (12.5)	972 (14.4)	
56-65	2300 (20.6)	813 (18.3)	1487 (22.1)	
66-75	2531 (22.7)	929 (20.9)	1602 (23.8)	
76-85	2370 (21.2)	969 (21.8)	1401 (20.8)	
>85	1153 (10.3)	598 (13.5)	555 (8.2)	
Ethnic group (n=9995, n=4003, n=5992, respectively)§:				
White	7457 (74.6)	3002 (75.0)	4455 (74.3)	0.12
Non-white	2538 (25.4)	1001 (25.0)	1537 (25.7)	
Country (n=10 866, n=4304, n=6562, respectively)§:				—
Belgium	234 (2.2)	88 (2.0)	146 (2.2)	
Egypt	44 (0.4%)	23 (0.5)	21 (0.3)	
Iran	83 (0.8)	30 (0.7)	53 (0.8)	
Italy	104 (1.0)	33 (0.8)	71 (1.1)	
Netherlands	4700 (43.3)	1729 (40.2)	2971 (45.3)	
Russia	307 (2.8)	151 (3.5)	156 (2.4)	
Saudi Arabia	381 (3.5)	117 (2.7)	264 (4.0)	
UK	4868 (44.8)	2085 (48.4)	2783 (42.4)	
Other countries†	1454 (1.3)	47 (1.1)	97 (1.5)	
**Vital signs at admission**				
Mean (SD) temperature (^o^C)	37.5 (1.1)	37.5 (1.1)	37.6 (1.1)	0.01
Median (IQI) respiratory rate (breaths/min)	20.0 (18.0-25.0)	20.0 (18.0-24.0)	21.0 (18.0-26.0)	0.01
Mean (SD) heart rate (beats/min)	90.7 (19.4)	90.8 (18.9)	90.8 (19.8)	0.95
Mean (SD) systolic blood pressure (mm Hg)	132.8 (22.8)	132.8 (23.3)	132.9 (22.5)	0.85
Mean (SD) diastolic blood pressure (mm Hg)	76.2 (14.2)	75.4 (14.5)	76.8 (14.0)	0.01
Median (IQI) oxygen saturation (%)	95.0 (93.0-97.0)	96.0 (93.0-98.0)	95.0 (92.0-97.0)	0.01
**Laboratory values at admission**				
Median (IQI) C reactive protein (mg/L)	76.0 (31.0-148.0)	63.0 (26.0-130.0)	85.0 (36.0-159.0)	0.01
Mean (SD) white blood cell count (×10^9^ /L)	8.0 (4.3)	7.8 (4.3)	8.1 (4.3)	0.01
Mean (SD) lymphocyte count (×10^9^ /L)	1.0 (0.8)	1.1 (0.8)	1.0 (0.8)	0.01
Mean (SD) haemoglobin (mmol/L)	8.1 (1.3)	7.7 (1.2)	8.4 (1.4)	0.01
Mean (SD) platelets (×10^9^ /L)	232.4 (107.7)	242.6 (107.7)	225.8 (107.1)	0.01
Mean (SD) creatinine (µmol/L)	103.9 (79.7)	90.7 (75.3)	112.3 (81.3)	0.01
**Cardiovascular risk factors**				
Mean (SD) body mass index	28.2 (5.9)	29.0 (6.8)	27.8 (5.1)	—
Median (IQI) body mass index	27.4 (24.2-31.2)	28.1 (24.1-32.8)	27.2 (24.3-30.5)	0.01
Body mass index group (n=7273, n=2759, n=4514, respectively)§:				—
<18.5	145 (2.0)	77 (2.8)	68 (1.5)	
18.5-24.9	2119 (29.1)	790 (28.6)	1329 (29.4)	
25.0-29.9	2674 (36.8)	827 (30.0)	1847 (40.9)	
30.0-34.9	1488 (20.5)	593 (21.5)	895 (19.8)	
>34.9	847 (11.6)	472 (17.1)	375 (8.3)	
Diabetes (n=11 008, n=4384, n=6624, respectively)§				
Yes	2863 (26.0)	1129 (25.8)	1734 (26.2)	0.29
Hypertension (n=10 896, n=4345, n=6551, respectively)§				
Yes	4951 (45.4)	2032 (46.8)	2919 (44.6)	0.01
Peripheral arterial disease (n=7269, n=2838, n=4431, respectively)§				
Yes	344 (4.7)	110 (3.9)	234 (5.3)	0.01
Dyslipidaemia (n=10 490, n=4168, n=6322, respectively)§				
Yes	3215 (30.6)	1155 (27.7)	2060 (32.6)	0.01
**History of cardiovascular disease‡**				
Any cardiovascular disease	3423 (33.9)	1217 (30.9)	2206 (35.9)	0.01
Arrhythmia/conduction disorder	1495 (14.8)	531 (13.5)	964 (15.7)	0.01
Supraventricular tachycardia	1222 (12.1)	456 (11.6)	766 (12.5)	0.01
Coronary artery disease	1416 (14.0)	348 (8.8)	1068 (17.4)	0.01
Myocardial infarction	722 (7.3)	163 (4.2)	559 (9.3)	0.01
Heart failure	732 (7.3)	312 (7.9)	420 (6.8)	0.01
Valvular heart disease	438 (4.3)	176 (4.5)	262 (4.3)	0.01
Congenital heart disease	40 (0.4)	19 (0.5)	21 (0.3)	0.01
Other cardiac disease	491 (4.9)	214 (5.4)	277 (4.5)	0.01

Values are numbers (percentages) unless stated otherwise.

IQI=interquartile interval.

*t test for continuous variables with a normal distribution; non-parametric Wilcoxon test for non-normal distribution; Pearson’s χ^2^ test for categorical variable.

†France, Israel, Portugal, and Spain.

‡Any cardiovascular disease: n=10 097 for all patients, n=3944 female participants, n=6153 male participants; supraventricular tachycardia: n=10 071, n=3931, n=6140; myocardial infarction: n=9913, n=3897, n=6016; all other cardiovascular disease subtypes: n=10 087, n=3939, n=6148.

§Some characteristics have varying totals for study groups (ie, all, female, and male participants, respectively).

### Complaints at admission

Median time from symptom onset to admission to hospital was four days (interquartile interval 1-8) and five days (1-9) in female and male participants, respectively. The most common symptoms in both sexes were fever, shortness of breath, and cough, each reported slightly less frequently in female than in male participants (2353/4396, 53% *v* 3881/6680, 58%; 2261/4396, 51% *v* 3691/6680, 55%; and 2224/4396, 50% *v* 3648/6680, 54%, respectively). Overall, the distribution of symptoms at admission was similar in both sexes, and in those aged ≤65 years and >65 years (online supplemental figure 3). Chest pain was reported by 9% (381/4396) of female participants and 8% (532/6680) of male participants, and 1% of female and male individuals (45/4396 and 67/6680, respectively) reported palpitations at admission. All recorded baseline measurements of vital signs and laboratory measurements were clinically or statistically similar (P≥0.05) between the sexes (online supplemental figure 4).

### Characteristics of hospital admission

Median length of hospital stay was eight days (interquartile interval 4-15) and nine days (4-17) in female and male participants, respectively. A smaller percentage of female than male participants were admitted to the intensive care unit (750/4438, 17% *v* 1781/6729, 27%) (online supplemental table 5). Among the 2531 individuals admitted to the intensive care unit, a smaller percentage of female than male participants received extracorporeal membrane oxygenation, vasopressor or inotropic support, or invasive or non-invasive ventilation.

### Sex differences in risk of cardiovascular complications and all cause mortality

**Figure 1:**
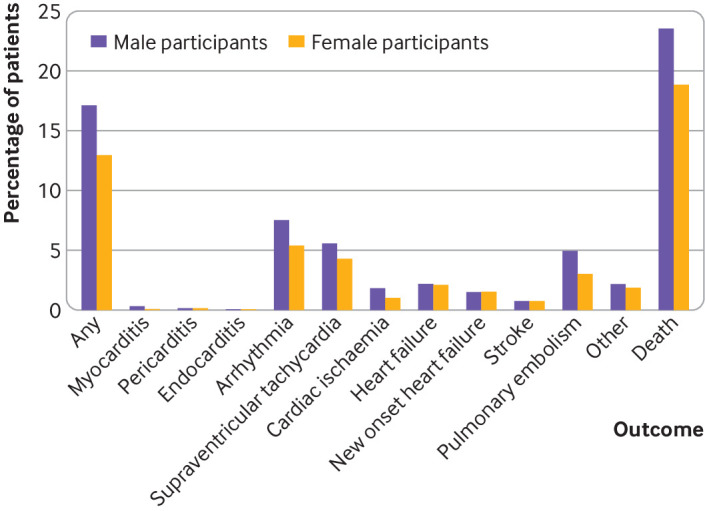
Any and specific cardiovascular complications in female and male participants admitted to hospital for covid-19

The risk of any cardiovascular complication was 13% (575/4438) and 17% (1152/6729) in female and male participants, respectively (figure 1). For both sexes, the most common complication was arrhythmia, in 5% of female participants (240/4438) and in 8% of male participants (507/6729), specifically supraventricular tachycardia (female individuals 191/4438, 4% *v* male individuals 376/6729, 6%), followed by pulmonary embolism (135/4438, 3% *v* 334/6729, 5%) and heart failure (94/4438, 2% *v* 149/6729, 2%).

**Figure 2:**
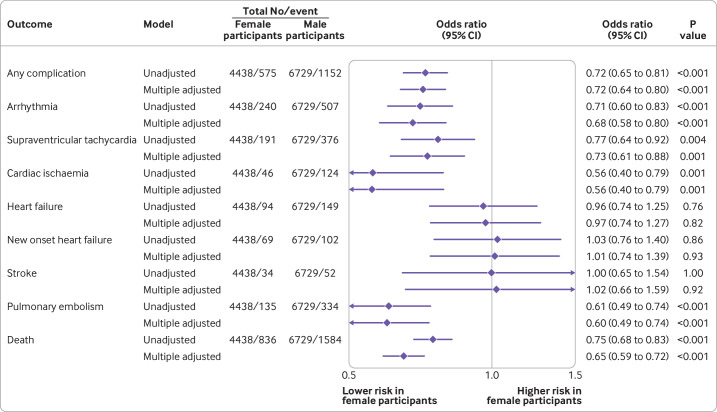
Odds ratios (95% confidence intervals) for the association between sex and cardiovascular outcomes. Unadjusted and adjusted estimates are presented. In adjusted analyses, models were adjusted for age, ethnic group, history of cardiovascular disease, use of relevant drug treatment for cardiovascular disease, and risk factors for cardiovascular disease (hypertension, diabetes, dyslipidaemia, peripheral arterial disease, and body mass index)

**Figure 3:**
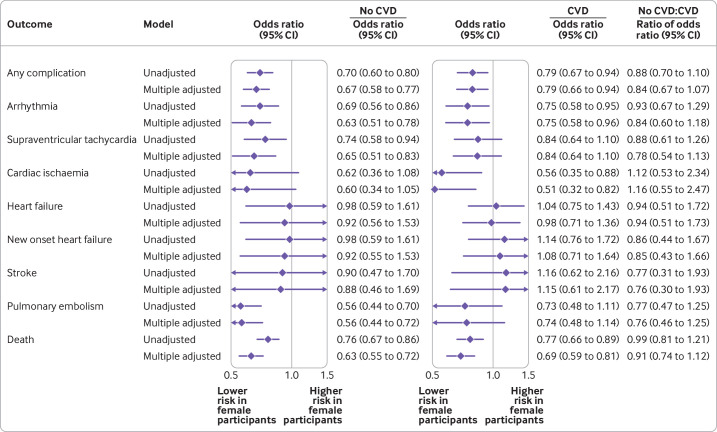
Female-to-male odds ratios (95% confidence intervals) in patients with no pre-existing cardiovascular disease (CVD) and in those with pre-existing CVD, and corresponding ratio of odds ratio (with 95% confidence interval). Unadjusted and adjusted estimates are presented. In adjusted analyses, models were adjusted for age, ethnic group, history of cardiovascular disease, use of relevant drug treatment for cardiovascular disease, and risk factors for cardiovascular disease (hypertension, diabetes, dyslipidaemia, peripheral arterial disease, and body mass index)

In analyses adjusted for age and ethnic group, female participants had a 29% lower odds than male participants of any cardiovascular complication (odds ratio 0.71, 95% confidence interval 0.63 to 0.79) (online supplemental figure 5). This association was similar after also adjusting for pre-existing cardiovascular disease, use of relevant drug treatment for cardiovascular disease, and risk factors for cardiovascular disease (odds ratio 0.72, 0.64 to 0.80) (figure 2). When grouped by pre-existing cardiovascular disease status, the female-to-male odds ratio was 0.67 (0.58 to 0.77) in patients with no pre-existing cardiovascular disease and 0.79 (0.66 to 0.94) in those with pre-existing cardiovascular disease (figure 3), with a ratio of odds ratio of 0.84 (0.67 to 1.07).

In analyses adjusted for age, ethnic group, pre-existing cardiovascular disease, use of drug treatments, and risk factors for cardiovascular disease, female participants were 35% less likely than male participants to die in hospital (odds ratio 0.65, 95% confidence interval 0.59 to 0.72). Female participants also had a lower risk of arrhythmia, supraventricular tachycardia, cardiac ischaemia, and pulmonary embolism than male participants (adjusted odds ratio 0.68 (0.58 to 0.80), 0.73 (0.61 to 0.88), 0.56 (0.40 to 0.79), and 0.60 (0.49 to 0.74), respectively). We found no differences between the sexes for the risk of heart failure or stroke (figure 2; online supplemental figure 5). In analyses grouped by pre-existing cardiovascular disease, point estimates for the ratio of odds ratio were all <1 (with the exception of cardiac ischaemia, where confidence intervals were wide). However, none was significantly different from 1 (figure 3).

### Sensitivity analyses

Complete case analyses showed similar patterns to the main analysis (online supplemental figures 6 and 7), and also when analyses were restricted to patients recruited at sites with universal or random sampling (online supplemental figures 8 and 9).

## Discussion

### Principal findings

In this registry based analysis of 11 167 adults admitted to hospital with covid-19 between March 2020 and May 2021, female sex was independently associated with a lower risk of any cardiovascular complication, any arrhythmia (including supraventricular tachycardia), supraventricular tachycardia, cardiac ischaemia, pulmonary embolism, and death; no difference was found for the risk of heart failure or stroke. Among the outcomes that showed an advantage for female individuals, the reduced risk could not be attributed to their lower prevalence of pre-existing cardiovascular disease; the associations between sex and the outcomes were the same after adjusting for pre-existing cardiovascular disease, and the difference in risk between the sexes was similar among those with and without pre-existing cardiovascular disease.

### Comparison with other studies

The literature on sex differences in the severity of covid-19 has largely focused on admissions to hospital and the intensive care unit, and death, universally showing a reduced risk in female individuals.^9 25 26^ A meta-analysis of 70 studies of 2 751 115 patients with covid-19 (those admitted to hospital as well as those in the community) and 214 361 deaths found that female sex was associated with a 28% lower odds of dying from covid-19.^9^ In patients with covid-19 who required admission to the intensive care unit and vital organ support, female participants were still 37% less likely than male participants to die in the intensive care unit, independent of age, severity of the acute critical illness, lifestyle factors, and comorbidities.^26^ In our study, female participants had a 35% lower odds of death than male participants while in hospital. Whether this difference between the sexes is the same or greater than would be expected in the general population, where male individuals have a shorter life expectancy, is unclear. For comparison, in the Global Burden of Disease Study, the age-standardised rate for all cause mortality in 2019 was 616 per 100 000 female individuals compared with 874 per 100 000 male individuals, corresponding to a female-to-male relative risk of about 0.70 (http://ghdx.healthdata.org/gbd-results-tool). For infections of the lower respiratory tract, the rate was 30 per 100 000 female individuals compared with 40 per 100 000 male individuals (relative risk 0.75). The difference in mortality between the sexes in our study was therefore greater than that for all cause mortality and other lower respiratory infections, but differences in the methodology between the studies need to be considered. In a study based on country level mortality data, where the magnitude of sex differences in age standardised covid-19 mortality was directly compared with that for all cause mortality and other common causes of death, a considerably greater sex difference for covid-19 was found.^25^ A better understanding of the extent to which reduced mortality in female individuals with covid-19 differs from already known sex differences in the non-COVID-19 setting is needed.

Cardiovascular complications during covid-19 were reported early in the pandemic, and sex differences in cardiovascular disease in general are known,^10 27–29^ but few studies have assessed sex differences across a range of cardiovascular complications associated with covid-19. Those studies that have reported sex differences are small,^30^ have described few cardiovascular events,^16^ or focused on one cardiovascular subtype,^31 32^ and have reported inconsistent findings showing either a lower risk of cardiovascular complications in female individuals or no difference between the sexes.^16 30 33^ We found that female sex was associated with a lower risk of any cardiovascular complication, any arrhythmia, supraventricular tachycardia, cardiac ischaemia, and pulmonary embolism, even after adjusting for pre-existing cardiovascular disease and risk factors for cardiovascular disease. Whether the advantage in female individuals and its magnitude is unique to covid-19 or whether similar patterns exist for other respiratory illnesses is unclear. In general, however, female individuals have been shown to have a lower age adjusted incidence of cardiovascular outcomes than male individuals, including atrial fibrillation^34^ and myocardial infarction.^10^

Pre-existing cardiovascular disease has also been repeatedly shown to be associated with worse covid-19 outcomes.^6 8 35^ In cardiovascular epidemiology, although female individuals generally have a lower risk of acute coronary syndrome and all cause mortality than male individuals, this advantage is considerably reduced after myocardial infarction.^10^ We investigated whether a similar interaction existed between pre-existing cardiovascular disease and sex for covid-19 disease (ie, whether pre-existing cardiovascular disease reduced the difference in the severity of covid-19 between the sexes). With coronary calcification as a marker of the risk of cardiovascular disease, previous research from Italy found that the protective effect in female individuals disappeared in those with moderate-to-severe coronary calcification, suggesting that a similar phenomenon might exist for covid-19.^16^ Grouped by pre-existing cardiovascular disease, in this study we found that the point estimate for the female-to-male odds ratio was smaller (indicating a greater advantage in female individuals) in people with no pre-existing cardiovascular disease than in those with cardiovascular disease for all outcomes except cardiac ischaemia. Although none of the ratio of odds ratios was significant, the consistency with which point estimates were reduced in those with pre-existing cardiovascular disease warrants further investigation.

Several candidate mechanisms to explain the sex differences in covid-19 have been suggested, with numerous reviews on the topic published.^14 36–38^ For example, differences between the sexes in the expression and activity of angiotensin converting enzyme 2 (ACE2), the SARS-CoV-2 receptor, and a key regulator of the renin-angiotensin system could feasibly have a role in directly influencing susceptibility to the SARS-CoV-2 virus in organs where ACE2 is expressed (including the heart),^39^ or in determining the extent of dysregulation of the renin-angiotensin system after ACE2 loss at the cell surface induced by SARS-CoV-2 infection.^14 37 38^ Although the relation between SARS-CoV-2, ACE2, and the renin-angiotensin system, and sex (and age) has yet to be fully elucidated, the evidence so far, from both covid-19 and cardiovascular disease in general, indicates that sex differences in both of these mechanisms are likely to be to the detriment of male individuals.^37 39^ Conversely, female individuals have been shown to have rapid and more effective innate and adaptive immune responses than male individuals, with male individuals more likely to develop systemic inflammation and the cytokine storm associated with worse covid-19 outcomes.^36–38^

Although our findings cannot clarify the plausibility and relative contribution of these various pathways to the differences in cardiovascular complications of covid-19 seen between the sexes, the persistence of the female advantage in those with pre-existing cardiovascular disease suggests that the pathophysiological mechanisms of covid-19 itself might differentially affect the sexes (rather than being an indication of general sex differences in the risk of cardiovascular disease). This finding is further supported by the absence of a difference in the risk of heart failure or stroke between the sexes in our study, in contrast with what is known for the general population^29 40^ Furthermore, although evidence from the non-covid-19 setting indicates that the lower risk of cardiovascular disease in female individuals might be lost or diminished after the menopause,^11 41–43^ we found clear sex differences in the risk of cardiovascular disease associated with covid-19 even though the median age of our cohort was 68 years. This finding has implications for future studies investigating the role of sex hormones in sex differences in covid-19.^38^

The complication profile of covid-19 was similar between the sexes. For example, although serious cardiac complications, such as myocarditis, pericarditis, endocarditis, and acute coronary syndrome, were rare in both sexes (≤0.5% for all), arrhythmia was the most common complication in both sexes. This finding suggests that similar treatment approaches to limiting the effect of covid-19 on cardiovascular health might be appropriate in both sexes. Female and male individuals have been reported to have different long term outcomes after myocarditis, cardiac arrest, and thrombotic events, independent of covid-19.^44^ Research is therefore needed to understand whether sex differences in the effect of covid-19 on cardiovascular health are worsened, reduced, or even reversed over time. Research is especially important given the findings from a large US study that found that patients who survived covid-19 had an increased risk of incident cardiovascular disease 12 months after infection, compared with those with no history of covid-19.^45^

Because our study involved patients who were admitted to hospital, which typically accounts for <10% of all confirmed patients with covid-19 disease,^46^ we assessed whether male participants arrived in more ill heath than female participants and so, once admitted to hospital, are more likely to have cardiovascular complications or die. We found no clinically meaningful sex differences in any of the measurements of vital signs or laboratory values at admission. This result is in line with previous findings showing persistent sex differences even after adjusting for the Acute Physiology and Chronic Health Evaluation II (APACHE II) score, indicating the severity of acute illness.^26^ Previous research, however, has shown that the association between peak levels of C reactive protein and adverse outcomes in covid-19 is stronger in male individuals.^47^ The higher risk of severe disease in male individuals among patients admitted to hospital for covid-19 might also be explained by their later presentation to hospital than female individuals. In this study, median time from symptom onset to admission was four days in female individuals and five days in male individuals; whether this difference is enough to affect the risk of cardiovascular complications and death from covid-19 during hospital admission is unclear.

### Strengths and limitations of this study

The strengths of our study include the large number of participants, which allowed us to examine sex differences for a range of cardiovascular outcomes. Our findings were limited to patients with covid-19 who were admitted to hospital, however, and therefore sex differences, if any, in the cardiovascular health of patients who recover from covid-19 without requiring admission to hospital is unclear. We also could not assess whether sex differences exist in cardiovascular complications or deaths that occurred outside of the hospital setting or in the long term outcomes of covid-19. Although the age distribution of our cohort was wide, with >30% of patients aged >75 years, generalising our findings to those who are very old or younger adults with cardiovascular complications associated with covid-19 is difficult. Furthermore, we acknowledge that the binary distinction between white and non-white ethnic groups did not capture the full spectrum of ethnic diversity in the population. Although other categories were captured in the registry (Asian, black, Latin American, and mixed ethnic groups), the percentage of participants was small. Therefore, for statistical reasons, and because the primary objective of the study was to examine sex differences, we chose to combine these groups. In the CAPACITY-COVID registry, pre-existing cardiovascular disease was derived from patients’ electronic health records and so misclassification of this exposure is possible. Central determination of complications did not exist.

Finally, the ever changing landscape of the covid-19 pandemic needs to be considered, in terms of the dominant variants in circulation, availability of effective treatments to minimise the effect of the disease on the body, and rates of vaccination, all of which could alter the relation between sex, covid-19, and cardiovascular disease. Data used in this study were collected between March 2020 and May 2021, when the dominant SARS-CoV-2 lineages in Europe were D614G followed by the alpha variant.^18–21^ Also, no vaccinations were available until December 2020, after which vaccination rates increased rapidly, with 60% and 40% of the population in the UK and the Netherlands, respectively, receiving their first dose by June 2021.^20 22 23^ Our findings therefore relate to when the direct effect of covid-19 was greatest. Nevertheless, we believe our findings have implications for our overall understanding of sex differences in health and disease, and show the importance of considering sex and gender differences across all aspects of human health.^48^

### Conclusions

Sex as an important disease modifier is increasingly recognised, including in covid-19, but there is still much we do not know about differences in covid-19 disease between the sexes, in particular whether the differences are greater than those seen in the non-covid-19 setting. Our findings suggest that differences in the severity of covid-19 between the sexes extend to cardiovascular complications of the disease and that these differences might not be explained by differences in the prevalence of pre-existing cardiovascular disease.

## Data Availability

Data are available upon reasonable request. The data used in this study might be available from the CAPACITY-COVID Collaborative Consortium. Researchers who are interested in investigating the role of cardiovascular disease in the covid-19 pandemic can apply for data access approval by the CAPACITY data access committee. The programming code developed for this study is available on reasonable request.
