# Supplementary material for: Sex differences in cardiovascular complications and mortality in hospital patients with covid-19: registry based observational study
Source: BMJ Med. 2023 Feb 14;2(1):e000245. doi: 10.1136/bmjmed-2022-000245 (PMC10083523; doi:10.1136/bmjmed-2022-000245)
Supplement: Supplementary data [file bmjmed-2022-000245supp001.pdf]

Supplementary Methods

Data cleaning, analysis and visualisation were performed using the following R packages: *mice* (version 3.13.0),[1] *arsenal* (version 3.6.3),[2] *dplyr* (version 1.0.6)[3] and *forestplot* (version 2.0.1)[4] packages.

Hockham C, *et al. BMJMED* 2023; 2:e000245. doi: 10.1136/bmjmed-2022-000245

Supplementary Table and Figure Legends

Supplementary Table 1 List of relevant medications or interventions for each outcome. Myocarditis, pericarditis, endocarditis and acute coronary syndrome were defined according to the diagnostic criteria of the corresponding European Society of Cardiology (ESC) guidelines.[5-8] For arrhythmias, definitions were based on the American College of Cardiology (ACC)/American Heart Association (AHA)/Heart Rhythm Society (HRS) 2006 data standards.[9]

Supplementary Table 2 Variables included in the multiple imputation model and their level of missingness, overall and by sex.

Supplementary Table 3 Percentage of participants receiving treatment with cardiovascular medications or procedures, by sex. Information on baseline medication use was missing for 0.1% of participants. The number of participants with missing information on prior procedures was 1080 (9.7%) overall, 499 (11.2%) in females and 581 (8.6%) in males. S = suppressed due to small numbers <20. PCI = percutaneous coronary intervention, CABG = coronary artery bypass graft.

Supplementary Table 4 Baseline characteristics of the CAPACITY-COVID registry participants, by sex and pre-existing cardiovascular disease (CVD).

Supplementary Table 5 Hospitalisation characteristics and outcomes of the CAPACITY-COVID registry participants, by sex.

Supplementary Figure 1 Recruitment to the CAPACITY-COVID registry over time.

Supplementary Figure 2 Flow diagram of cohort selection.

Supplementary Figure 3 Presenting symptoms in females and males, stratified by age (top panel: ≤65 years; bottom panel: >65 years). Orange = females; Green = males. GI = gastrointestinal.

Supplementary Figure 4 Summary statistics of vital measurements (temperature, respiratory rate, heart rate, blood pressure and oxygen saturation) and laboratory measurements (C-reactive protein, white blood cell count, lymphocyte count, haemoglobin, platelets and creatinine), by sex.

Supplementary Figure 5 Odds ratios (ORs, with 95% confidence intervals [CIs]) for the association between sex and the outcomes, using four sets of model adjustments: (i) no adjustment, (ii) age and ethnicity, (iii) age, ethnicity, cardiovascular disease (CVD) history and medication use, and (iv) age, ethnicity, CVD history, medication use and CVD risk factors (hypertension, diabetes, dyslipidemia, peripheral arterial disease and body mass index).

Supplementary Figure 6 Complete case analyses: Odds ratios (ORs, with 95% confidence intervals [CIs]) for the association between sex and the outcomes of interest. Unadjusted and adjusted estimates are presented. In adjusted analyses, models were adjusted for age, ethnicity, cardiovascular disease (CVD) history, medication use and CVD risk factors (hypertension, diabetes, dyslipidemia, peripheral arterial disease and body mass index).

Supplementary Figure 7 Complete case analyses: Female-to-male odds ratios (ORs, with 95% confidence intervals [CIs]) in the cohorts without and with pre-existing cardiovascular disease (CVD), and the corresponding ratio of OR (with 95% CI). Unadjusted and adjusted estimates are presented. In adjusted analyses, models were adjusted for age, ethnicity, CVD history, medication use and CVD risk factors (hypertension, diabetes, dyslipidemia, peripheral arterial disease and body mass index).

Supplementary Figure 8 Sensitivity analyses with patients from selective recruitment sites excluded: Odds ratios (ORs, with 95% confidence intervals [CIs]) for the association between sex and the outcomes of interest. Unadjusted and adjusted estimates are presented. In adjusted analyses, models

Hockham C, *et al*. *BMJMED* 2023; 2:e000245. doi: 10.1136/bmjmed-2022-000245

were adjusted for age, ethnicity, cardiovascular disease (CVD) history, medication use and CVD risk factors (hypertension, diabetes, dyslipidemia, peripheral arterial disease and body mass index).

Supplementary Figure 9 Sensitivity analyses with patients from selective recruitment sites excluded: Female-to-male odds ratios (ORs, with 95% confidence intervals [CIs]) in the cohorts without and with pre-existing cardiovascular disease (CVD), and the corresponding ratio of OR (with 95% CI). Unadjusted and adjusted estimates are presented. In adjusted analyses, models were adjusted for age, ethnicity, CVD history, medication use and CVD risk factors (hypertension, diabetes, dyslipidemia, peripheral arterial disease and body mass index).

Hockham C, *et al. BMJMED* 2023; 2:e000245. doi: 10.1136/bmjmed-2022-000245

Supplementary Material

Supplementary Table 1 List of relevant medications or interventions for each outcome. Myocarditis, pericarditis, endocarditis and acute coronary syndrome were defined according to the diagnostic criteria of the corresponding European Society of Cardiology (ESC) guidelines.[5-8] For arrhythmias, definitions were based on the American College of Cardiology (ACC)/American Heart Association (AHA)/Heart Rhythm Society (HRS) 2006 data standards.[9]

Cardiovascular complication (outcome)	Relevant medication/intervention
Any cardiac/thromboembolic complication / death	Any of:<br>Beta-blocker, antiarrhythmic drugs, digoxin, diuretics, CCBs, ACEIs, ARBs, aldosterone antagonists, sacubitril/valsartan, phosphodiesterase inhibitors, anti-platelet agents, coumarin, DOACs, lipid-lowering agents, other CV medications, CABG, PCI, and intervention for valvular heart disease performed<br><br>NOT: insulin/oral antidiabetic agents
Arrhythmia / supraventricular tachycardia	Any of:<br>Beta-blocker, Antiarrhythmic drugs, digoxin, and CCBs
Cardiac ischemia	Any of:<br>Beta-blocker, ACEIs, ARBs, anti-platelet agents, coumarin, DOACs, lipid-lowering agents,<br>Pharmacological intervention, CABG and PCI
Heart failure / new-onset heart failure	Any of:<br>Digoxin, diuretics, ACEIs, ARBs, aldosterone antagonists, sacubitril/valsartan, antiarrhythmic drugs
Stroke	Any of:<br>Anti-platelet agents, DOACs, coumarin
Pulmonary embolism	Any of :<br>Coumarin, DOACs, anti-platelet agents

Supplementary Table 2 Variables included in the multiple imputation model and their level of missingness, overall and by sex.

	n (%) missing		
**Characteristic**	**Total (n = 11167)**	**Female (n = 4438)**	**Male (n = 6729)**
**Demographic characteristics**			
Age	-	-	-
Ethnicity	1172 (10.5%)	435 (9.8%)	737 (11.0%)
**Cardiovascular disease history**			
Any cardiovascular disease	1070 (9.6%)	494 (11.1%)	576 (8.6%)
Arrhythmia/conduction disorder	1080 (9.7%)	499 (11.2%)	581 (8.6%)
Supraventricular tachycardia	1096 (9.8%)	507 (11.4%)	589 (8.8%)
Coronary artery disease	1080 (9.7%)	499 (11.2%)	581 (8.6%)
Myocardial infarction	1254 (11.2%)	541 (12.2%)	713 (10.6%)
Heart failure	1080 (9.7%)	499 (11.2%)	581 (8.6%)
Valvular heart disease	1080 (9.7%)	499 (11.2%)	581 (8.6%)
Congenital heart disease	1080 (9.7%)	499 (11.2%)	581 (8.6%)
Other cardiac disease	1080 (9.7%)	499 (11.2%)	581 (8.6%)
**Relevant CVD medication use**			
Any cardiovascular complication	284 (2.5%)	117 (2.6%)	167 (2.5%)
Arrhythmia / supraventricular tachycardia	2781 (24.9%)	1074 (24.2%)	1707 (25.4%)
Cardiac ischemia	844 (7.6%)	392 (8.8%)	452 (6.7%)
Heart failure	2657 (23.8%)	1029 (23.2%)	1628 (24.2%)
Stroke	3582 (32.1%)	1561 (35.2%)	2021 (30.0%)
Pulmonary embolism	3582 (32.1%)	1561 (35.2%)	2021 (30.0%)
**Cardiovascular risk factors**			
BMI	3894 (34.9%)	1679 (37.8%)	2215 (32.9%)
Diabetes	159 (1.4%)	54 (1.2%)	105 (1.6%)
Hypertension	271 (2.4%)	93 (2.1%)	178 (2.6%)
Peripheral arterial disease	3898 (34.9%)	1600 (36.1%)	2298 (34.2%)
Dyslipidemia	677 (6.1%)	270 (6.1%)	407 (6.0%)
**Outcomes**			
Any complication	104 (0.9%)	44 (1.0%)	60 (0.9%)
Arrhythmia	104 (0.9%)	44 (1.0%)	60 (0.9%)
Supraventricular tachycardia	112 (1.0%)	48 (1.1%)	64 (1.0%)
Cardiac ischemia	104 (0.9%)	44 (1.0%)	60 (0.9%)
Heart failure	104 (0.9%)	44 (1.0%)	60 (0.9%)
New-onset heart failure	104 (0.9%)	44 (1.0%)	60 (0.9%)
Stroke	104 (0.9%)	44 (1.0%)	60 (0.9%)
Pulmonary embolism	104 (0.9%)	44 (1.0%)	60 (0.9%)
All-cause mortality	55 (0.5%)	19 (0.4%)	36 (0.5%)

Hockham C, *et al. BMJMED* 2023; 2:e000245. doi: 10.1136/bmjmed-2022-000245

Supplementary Table 3 Percentage of participants receiving treatment with cardiovascular medications or procedures, by sex. Information on baseline medication use was missing for 0.1% of participants. The number of participants with missing information on prior procedures was 1080 (9.7%) overall, 499 (11.2%) in females and 581 (8.6%) in males. S = suppressed due to small numbers <20. PCI = percutaneous coronary intervention, CABG = coronary artery bypass graft.

	Total (n = 11167)	Female (n = 4438)	Male (n = 6729)
**Medication**			
Beta-blocker	2669 (23.9%)	1065 (24.0%)	1604 (23.9%)
Anti-arrhythmia agent	479 (4.3%)	184 (4.2%)	295 (4.4%)
Digoxin	210 (1.9%)	100 (2.3%)	110 (1.6%)
Diuretic	2054 (18.4%)	913 (20.6%)	1141 (17.0%)
Calcium channel blocker	1756 (15.7%)	699 (15.8%)	1057 (15.7%)
ACE inhibitor	1740 (15.6%)	584 (13.2%)	1156 (17.2%)
Angiotensin receptor blocker	1119 (10.0%)	468 (10.6%)	651 (9.7%)
Aldosterone antagonist	310 (2.8%)	123 (2.8%)	187 (2.8%)
Entresto	34 (0.3%)	S	S
Phosphodiesterase inhibitors	25 (0.2%)	S	S
Anti-platelet agent	1915 (17.2%)	636 (14.4%)	1279 (19.0%)
Coumarin	393 (3.5%)	136 (3.1%)	257 (3.8%)
Direct oral anticoagulant agent	1066 (9.6%)	408 (9.2%)	658 (9.8%)
Lipid-lowering agent	3588 (32.2%)	1275 (28.8%)	2313 (34.4%)
Insulin	806 (7.2%)	327 (7.4%)	479 (7.1%)
Oral anti-diabetic agent	1615 (14.5%)	611 (13.8%)	1004 (14.9%)
Other cardiovascular medication	493 (4.4%)	184 (4.2%)	309 (4.6%)
**Procedures**			
PCI	643 (6.4%)	142 (3.6%)	501 (8.1%)
CABG	376 (3.7%)	57 (1.4%)	319 (5.2%)
Intervention performed/planned	n = 438	n = 176	n = 262
No intervention	230 (52.5%)	110 (62.5%)	120 (45.8%)
Intervention	177 (40.4%)	50 (28.4%)	127 (48.5%)
Not specified	31 (7.1%)	16 (9.1%)	15 (5.7%)

Hockham C, *et al. BMJMED* 2023; 2:e000245. doi: 10.1136/bmjmed-2022-000245

Supplementary Table 4 Baseline characteristics of the CAPACITY-COVID registry participants, by sex and pre-existing cardiovascular disease (CVD).

	**Without pre-existing cardiovascular disease**			**With pre-existing cardiovascular disease**		
	**Total (n = 6674)**	**Female (n = 2727)**	**Male (n = 3947)**	**Total (n = 3423)**	**Female (n = 1217)**	**Male (n = 2206)**
**Demographic characteristics**						
Age (years), mean (SD)	62.3 (15.9)	63.5 (16.9)	61.6 (15.2)	74.6 (12.0)	76.1 (12.6)	73.8 (11.5)
Age group (years)						
18 - 25	100 (1.5%)	48 (1.8%)	52 (1.3%)	S	S	S
26 - 35	306 (4.6%)	146 (5.4%)	160 (4.1%)	19 (0.6%)	S	11 (0.5%)
36 - 45	564 (8.5%)	214 (7.8%)	350 (8.9%)	48 (1.4%)	25 (2.1%)	23 (1.0%)
46 - 55	1182 (17.7%)	423 (15.5%)	759 (19.2%)	167 (4.9%)	61 (5.0%)	106 (4.8%)
56 - 65	1643 (24.6%)	607 (22.3%)	1036 (26.2%)	452 (13.2%)	122 (10.0%)	330 (15.0%)
66 - 75	1388 (20.8%)	561 (20.6%)	827 (21.0%)	951 (27.8%)	282 (23.2%)	669 (30.3%)
76 - 85	1048 (15.7%)	479 (17.6%)	569 (14.4%)	1151 (33.6%)	414 (34.0%)	737 (33.4%)
>85	443 (6.6%)	249 (9.1%)	194 (4.9%)	632 (18.5%)	305 (25.1%)	327 (14.8%)
Ethnicity	n = 6013	n = 2476	n = 3937	n = 3195	n = 1141	n = 2054
White	4331 (72.0%)	1841 (74.4%)	2490 (70.4%)	2702 (84.5%)	961 (84.2%)	1741 (84.8%)
Non-White	1682 (28.0%)	635 (25.6%)	1 (29.6%)	493 (15.4%)	180 (15.8%)	313 (15.2%)
Country	n = 6472	n = 2629	n = 3843	n = 3324	n = 1181	n = 2143
Belgium	123 (1.9%)	49 (1.9%)	74 (1.9%)	110 (3.3%)	39 (3.3%)	71 (3.3%)
Egypt	21 (0.3%)	11 (0.4%)	10 (0.3%)	22 (0.7%)	11 (0.9%)	11 (0.5%)
Iran	14 (0.2%)	S	S	69 (2.1%)	22 (1.9%)	47 (2.2%)
Italy	20 (0.3%)	S	S	84 (2.5%)	29 (2.5%)	55 (2.6%)
Netherlands	3036 (46.9%)	1210 (46.0%)	1826 (47.5%)	1650 (49.6%)	513 (43.4%)	1137 (53.1%)
Russian Federation	188 (2.9%)	89 (3.4%)	99 (2.6%)	118 (3.5%)	61 (5.2%)	57 (2.7%)
Saudi Arabia	333 (5.1%)	94 (3.6%)	239 (6.2%)	47 (1.4%)	22 (1.9%)	25 (1.2%)
United Kingdom	2640 (40.8%)	1131 (43.0%)	1509 (39.3%)	1178 (35.4%)	470 (39.8%)	708 (33.0%)
Other*	97 (1.5%)	33 (1.3%)	64 (1.7%)	46 (1.4%)	14 (1.2%)	32 (1.5%)
**Cardiovascular risk factors**						
BMI (kg/m²), mean (SD)	28.4 (5.9)	29.2 (6.9)	27.9 (5.2)	27.9 (5.6)	28.4 (6.6)	27.7 (5.0)
BMI (kg/m²), median (IQI)	27.7 (24.4-31.4)	28.4 (24.2-32.9)	27.2 (24.4-30.6)	27.1 (24.2-30.9)	27.5 (23.8-32.0)	27.1 (24.4-30.4)
BMI group (kg/m²)	n = 6674	n = 1667	n = 2511	n = 2375	n = 786	n = 1589
<18.5	83 (2.0%)	46 (2.8%)	37 (1.5%)	51 (2.1%)	27 (3.4%)	24 (1.5%)
18.5 - 24.9	1190 (28.5%)	453 (27.2%)	737 (29.4%)	699 (29.4%)	244 (31.0%)	455 (28.6%)
25.0 - 29.9	1532 (36.7%)	513 (30.8%)	1019 (40.6%)	913 (38.4%)	232 (29.5%)	681 (42.9%)

30.0 - 34.9	868 (20.8%)	364 (21.8%)	504 (20.1%)	473 (19.9%)	164 (20.9%)	309 (19.4%)
>34.9	505 (12.1%)	291 (17.5%)	214 (8.5%)	239 (10.1%)	119 (15.1%)	120 (7.5%)
Diabetes	n = 6563	n = 2684	n = 3879	n = 3378	n = 1207	n = 2171
Yes	1482 (22.6%)	627 (23.4%)	855 (22.0%)	1190 (35.2%)	420 (34.8%)	770 (35.5%)
Hypertension	n = 6507	n = 2663	n = 3844	n = 3324	n = 1189	n = 2135
Yes	2446 (37.6%)	1079 (40.5%)	1367 (35.6%)	2235 (67.2%)	826 (69.5%)	1409 (66.0%)
Peripheral arterial disease	n = 4876	n = 1984	n = 2892	n = 2374	n = 847	n = 1527
Yes	106 (2.2%)	33 (1.7%)	73 (2.5%)	233 (9.8%)	75 (8.9%)	158 (10.3%)
Dyslipidemia	n = 6239	n = 2551	n = 3688	n = 3187	n = 1126	n = 2061
Yes	1441 (23.1%)	580 (22.7%)	861 (23.3%)	1661 (51.1%)	529 (47.0%)	1132 (54.9%)
**Cardiovascular disease history**						
Any cardiovascular disease	-	-	-	3423 (100.0%)	1217 (100.0%)	2206 (100.0%)
Arrhythmia/conduction disorder	-	-	-	1495 (43.8%)	531 (43.8%)	964 (43.8%)
Supraventricular tachycardia	-	-	-	1222 (36.0%)	456 (37.9%)	766 (34.9%)
Coronary artery disease	-	-	-	1416 (41.5%)	348 (28.7%)	1068 (48.5%)
Myocardial infarction	-	-	-	722 (22.3%)	163 (13.9%)	559 (27.0%)
Heart failure	-	-	-	732 (21.4%)	312 (25.7%)	420 (19.1%)
Stage of heart failure				n = 402	n = 166	n = 236
Stage I/II	-	-	-	179 (44.5%)	63 (38.0%)	116 (49.2%)
Stage III/IV	-	-	-	91 (22.6%)	37 (22.3%)	54 (22.9%)
Not specified	-	-	-	132 (32.8%)	66 (39.8%)	66 (28.0%)
Valvular heart disease	-	-	-	438 (12.8%)	176 (14.5%)	262 (11.9%)
Congenital heart disease	-	-	-	40 (1.2%)	19 (1.6%)	21 (1.0%)
Other cardiac disease	-	-	-	491 (14.4%)	214 (17.7%)	277 (12.6%)
**Medication use**[‡]	n = 6667	n = 2724	n = 3943	n = 3420	n = 1215	n = 2205
Beta-blocker	734 (11.0%)	373 (13.7%)	361 (9.2%)	1853 (54.2%)	657 (54.1%)	1196 (54.2%)
Anti-arrhythmia agent	95 (1.4%)	36 (1.3%)	59 (1.5%)	274 (8.0%)	92 (7.6%)	182 (8.3%)
Digoxin	29 (0.4%)	15 (0.6%)	14 (0.4%)	177 (5.2%)	82 (6.7%)	95 (4.3%)
Diuretic	755 (11.3%)	372 (13.7%)	383 (9.7%)	1214 (35.5%)	501 (41.2%)	713 (32.3%)
Calcium channel blocker	915 (13.7%)	387 (14.2%)	528 (13.4%)	747 (21.8%)	270 (22.2%)	477 (21.6%)
ACE inhibitor	716 (10.7%)	285 (10.5%)	431 (10.9%)	959 (28.0%)	273 (22.5%)	686 (31.1%)
Angiotensin receptor blocker	534 (8.0%)	239 (8.8%)	295 (7.5%)	550 (16.1%)	213 (17.5%)	337 (15.3%)
Aldosterone antagonist	65 (1.0%)	34 (1.2%)	31 (0.8%)	232 (6.8%)	83 (6.8%)	149 (6.8%)
Entresto	S	S	S	30 (0.9%)	S	24 (1.1%)
Phosphodiesterase inhibitors	15 (0.2%)	S	S	S	S	S

Anti-platelet agent	657 (9.9%)	273 (10.0%)	384 (9.7%)	1210 (35.4%)	349 (28.7%)	861 (39.0%)
Coumarin	45 (0.7%)	18 (0.7%)	27 (0.7%)	344 (10.1%)	118 (9.7%)	226 (10.2%)
Direct oral anticoagulant agent	241 (3.6%)	102 (3.7%)	139 (3.5%)	803 (23.5%)	296 (24.4%)	507 (23.0%)
Lipid-lowering agent	1579 (23.7%)	643 (23.6%)	936 (23.7%)	1857 (54.3%)	573 (47.2%)	1284 (58.2%)
Insulin	398 (6.0%)	170 (6.2%)	228 (5.8%)	342 (10.0%)	126 (10.4%)	216 (9.8%)
Oral anti-diabetic agent	864 (13.0%)	362 (13.3%)	502 (12.7%)	668 (19.5%)	209 (17.2%)	459 (20.8%)
Other cardiovascular medication	97 (1.5%)	41 (1.5%)	56 (1.4%)	396 (11.6%)	143 (11.8%)	253 (11.5%)
**Procedures**						
PCI	-	-	-	643 (6.4%)	142 (3.6%)	501 (8.1%)
CABG	-	-	-	376 (3.7%)	57 (1.4%)	319 (5.2%)
Intervention performed/planned				n = 438	n = 176	n = 262
No intervention	-	-	-	230 (52.5%)	110 (62.5%)	120 (45.8%)
Intervention	-	-	-	177 (40.4%)	50 (28.4%)	127 (48.5%)
Not specified	-	-	-	31 (7.1%)	16 (9.1%)	15 (5.7%)

*Other countries: France, Israel, Portugal, Spain

†Supraventricular tachycardia: n total = 3397, n female = 1204, n male = 2193; Myocardial infarction: n total = 3239, n female = 1170, n male = 2069; All other cardiovascular disease (CVD) sub-types: n total = 3413, n female = 1212, n male = 2201.

‡Information on baseline medication use was missing for 0.1% of participants without pre-existing CVD and 0.1% of participants with pre-existing CVD. The number of participants in the CVD cohort with missing information on prior procedures was 10 (0.4%) overall, 5 (0.2%) in females and 5 (0.4%) in males.

Supplementary Table 5 Hospitalisation characteristics and outcomes of the CAPACITY-COVID registry participants, by sex.

Characteristics	Total (n = 11167)	Female (n = 4438)	Male (n = 6729)
**Admission**			
Duration of hospitalisation (days), median (IQI)	8 (4-16)	8 (4-15)	9 (4-17)
Admission to intensive care unit (ICU)	2,531 (22.7%)	750 (16.9%)	1,781 (26.5%)
Time to ICU admission (days), median (IQI)	1 (0-4)*	1 (0-4)*	1 (0-4)*
Duration of stay in ICU (days), median (IQI)	12 (6-22)	11 (5-19)	12 (6-23)
**In-hospital treatment**	**(n = 2531)**	**(n = 750)**	**(n = 1781)**
Vasopressor/inotropic support	1055 (41.7%)	271 (36.1%)	784 (44.0%)
Invasive ventilation	1977 (78.1%)	563 (75.1%)	1,414 (79.4%)
Non-invasive ventilation	1316 (52.0%)	368 (49.1%)	948 (53.2%)
ECMO	119 (4.7%)	26 (3.5%)	93 (5.2%)

*n total = 2272, n female = 678, n male = 1594

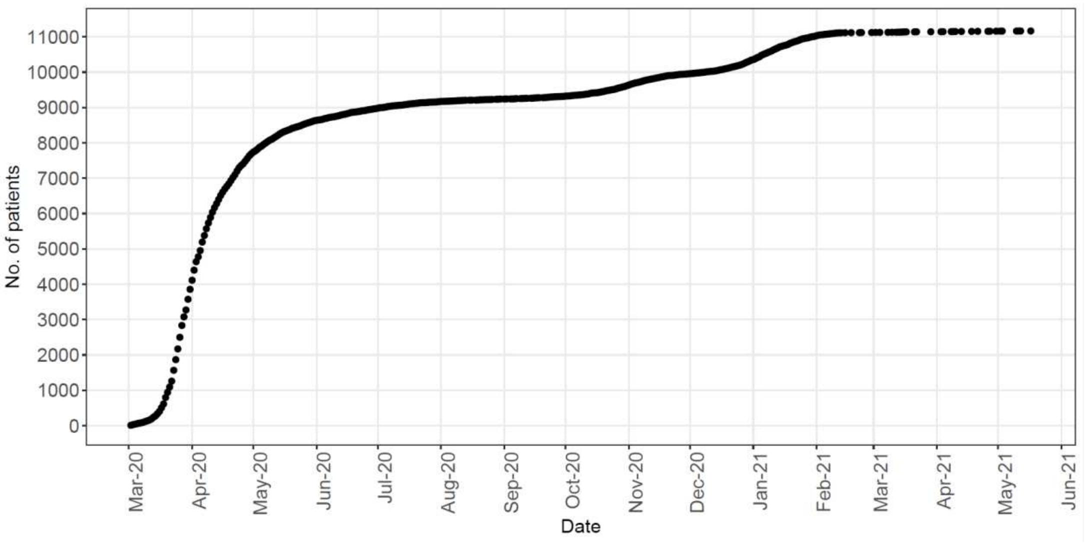

Supplementary Figure 1 Recruitment to the CAPACITY-COVID registry over time.

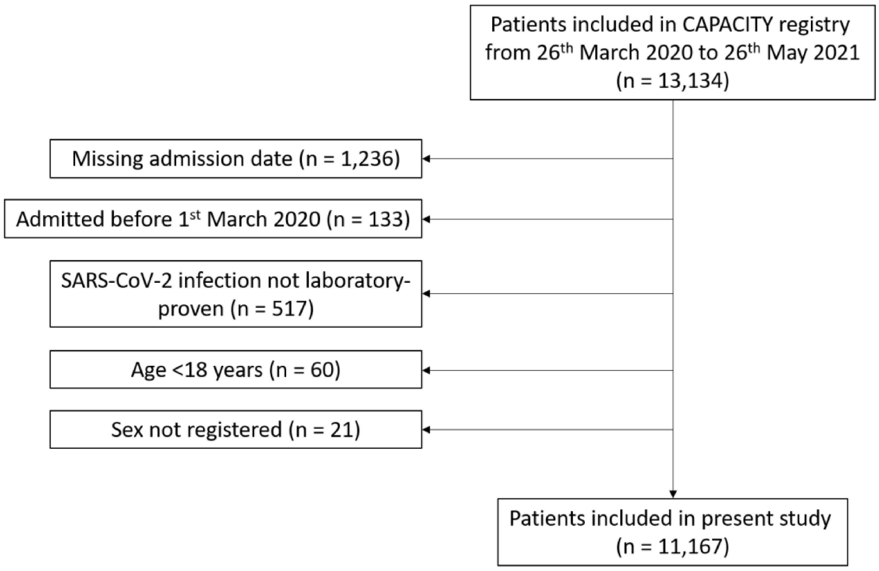

Supplementary Figure 2 Flow diagram of cohort selection.

Hockham C, *et al. BMJMED* 2023; 2:e000245. doi: 10.1136/bmjmed-2022-000245

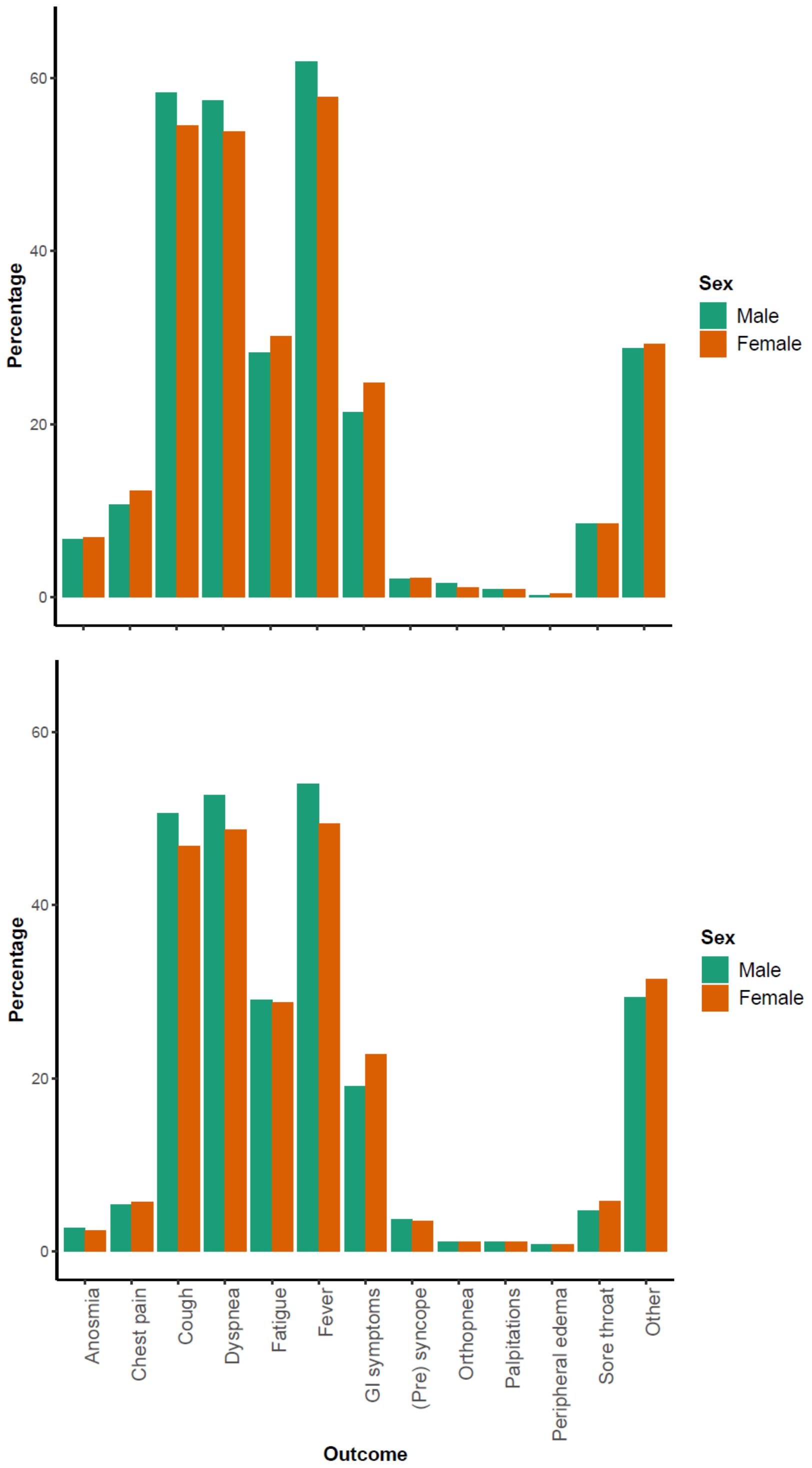

Supplementary Figure 3 Presenting symptoms in females and males, stratified by age (top panel: ≤65 years; bottom panel: >65 years). Orange = females; Green = males. GI = gastrointestinal.

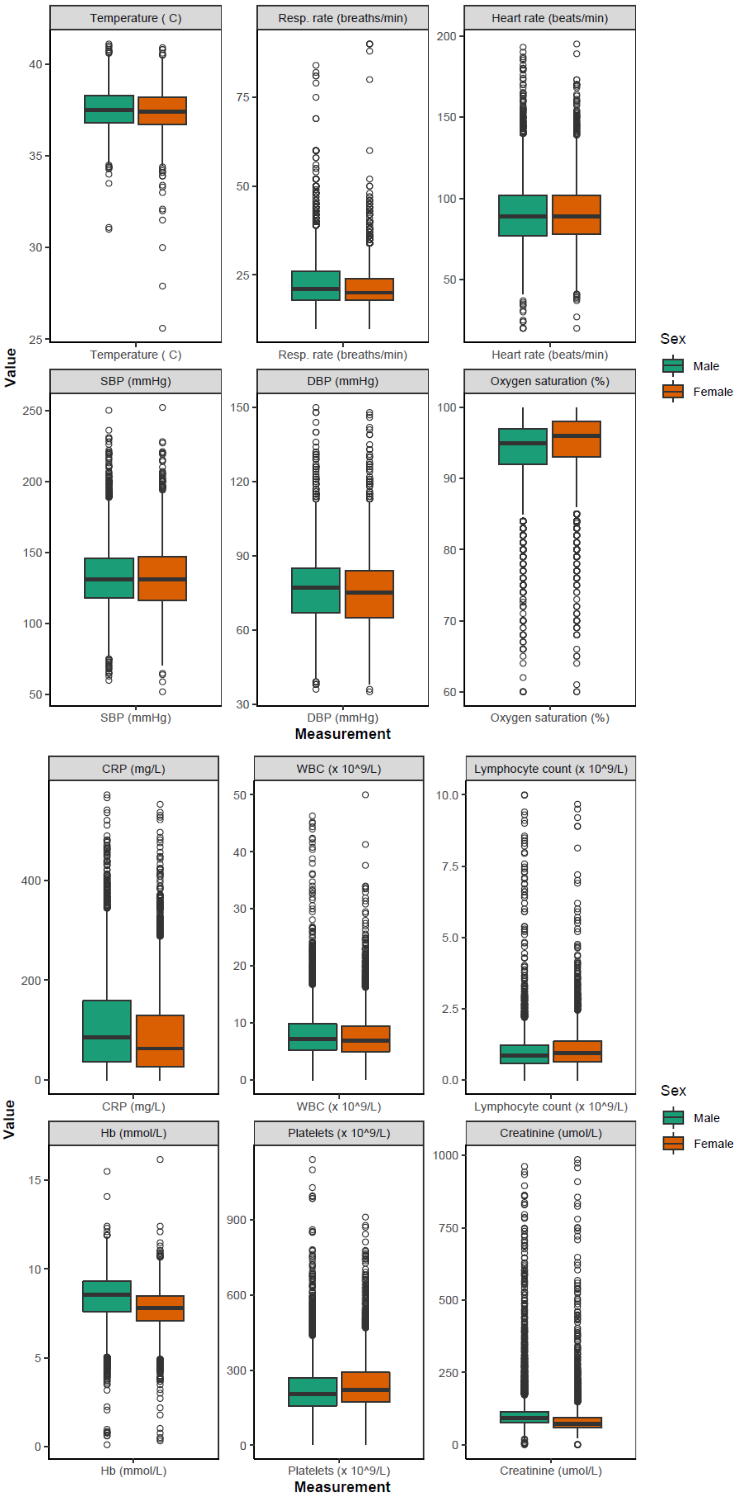

Supplementary Figure 4 Summary statistics of vital measurements (temperature, respiratory rate, heart rate, blood pressure and oxygen saturation) and laboratory measurements (C-reactive protein, white blood cell count, lymphocyte count, haemoglobin, platelets and creatinine), by sex.

Hockham C, *et al. BMJMED* 2023; 2:e000245. doi: 10.1136/bmjmed-2022-000245

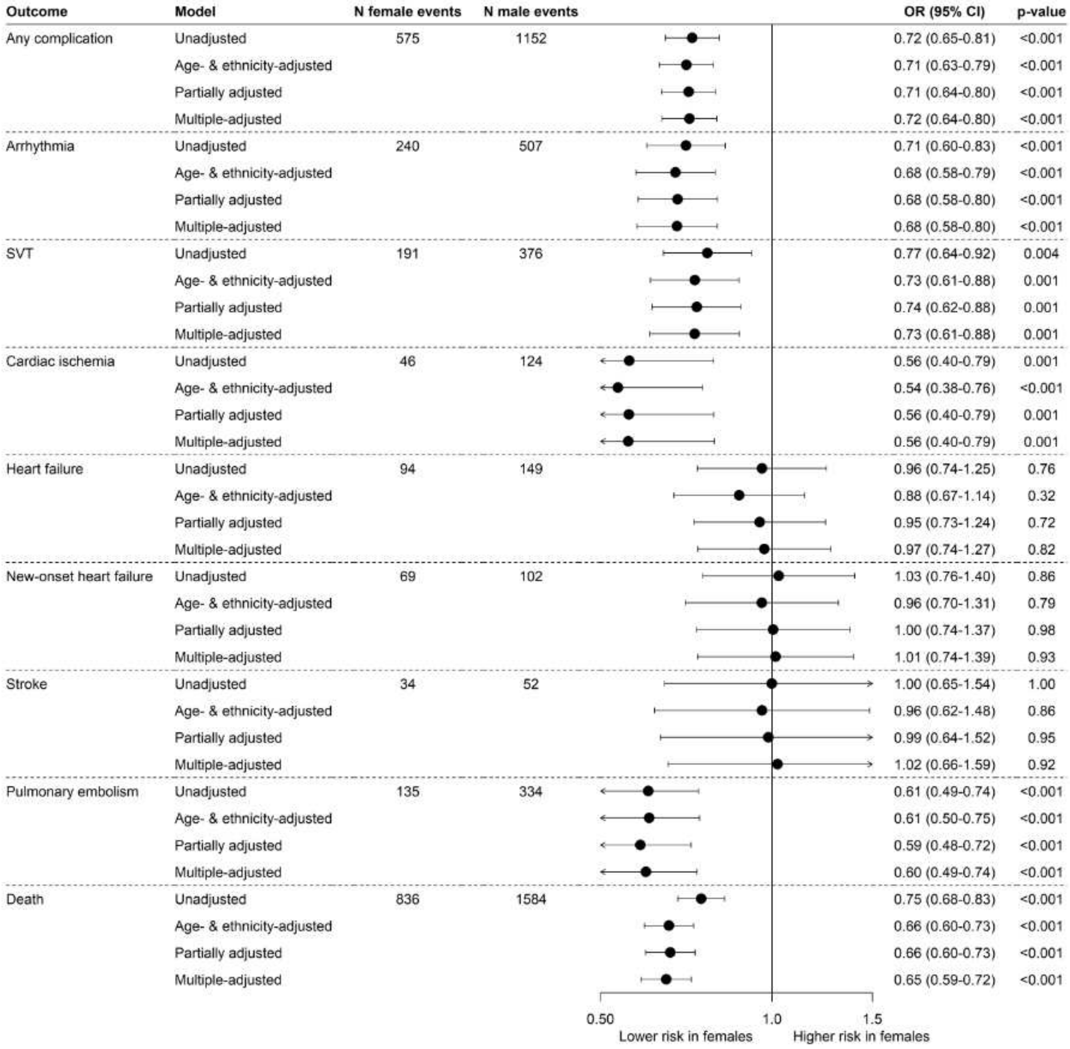

Supplementary Figure 5 Odds ratios (ORs, with 95% confidence intervals [CIs]) for the association between sex and the outcomes, using four sets of model adjustments: (i) no adjustment, (ii) age and ethnicity, (iii) age, ethnicity, cardiovascular disease (CVD) history and medication use, and (iv) age, ethnicity, CVD history, medication use and CVD risk factors (hypertension, diabetes, dyslipidemia, peripheral arterial disease and body mass index).

Hockham C, *et al. BMJMED* 2023; 2:e000245. doi: 10.1136/bmjmed-2022-000245

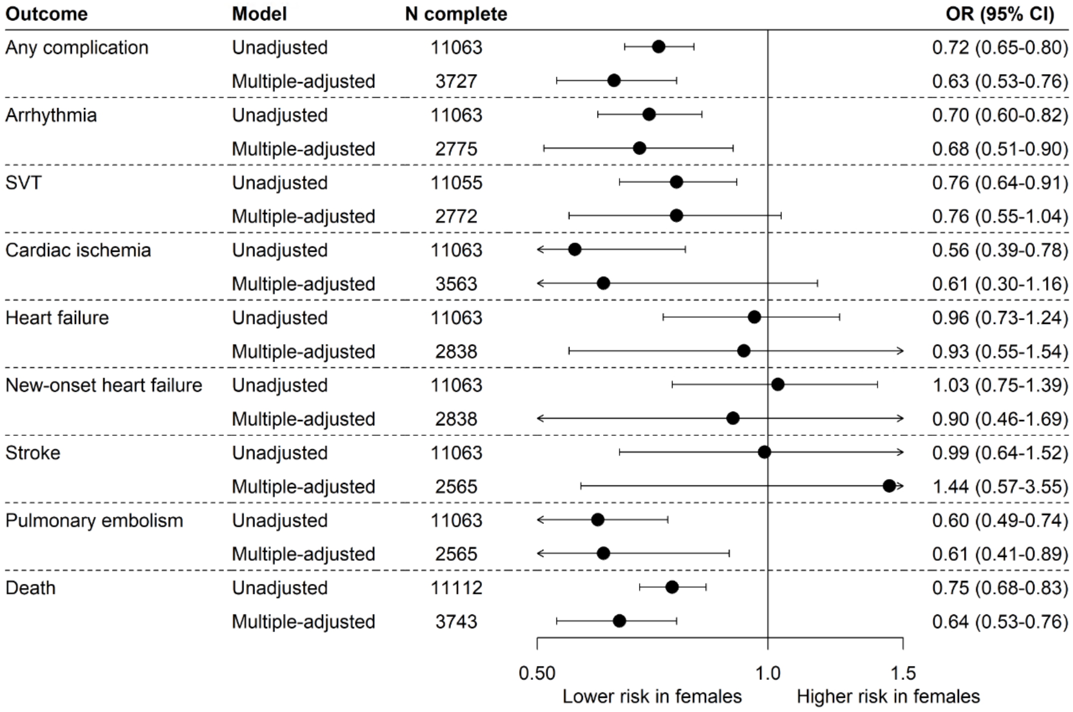

Supplementary Figure 6 Complete case analyses: Odds ratios (ORs, with 95% confidence intervals [CIs]) for the association between sex and the outcomes of interest. Unadjusted and adjusted estimates are presented. In adjusted analyses, models were adjusted for age, ethnicity, cardiovascular disease (CVD) history, medication use and CVD risk factors (hypertension, diabetes, dyslipidemia, peripheral arterial disease and body mass index).

Hockham C, *et al. BMJMED* 2023; 2:e000245. doi: 10.1136/bmjmed-2022-000245

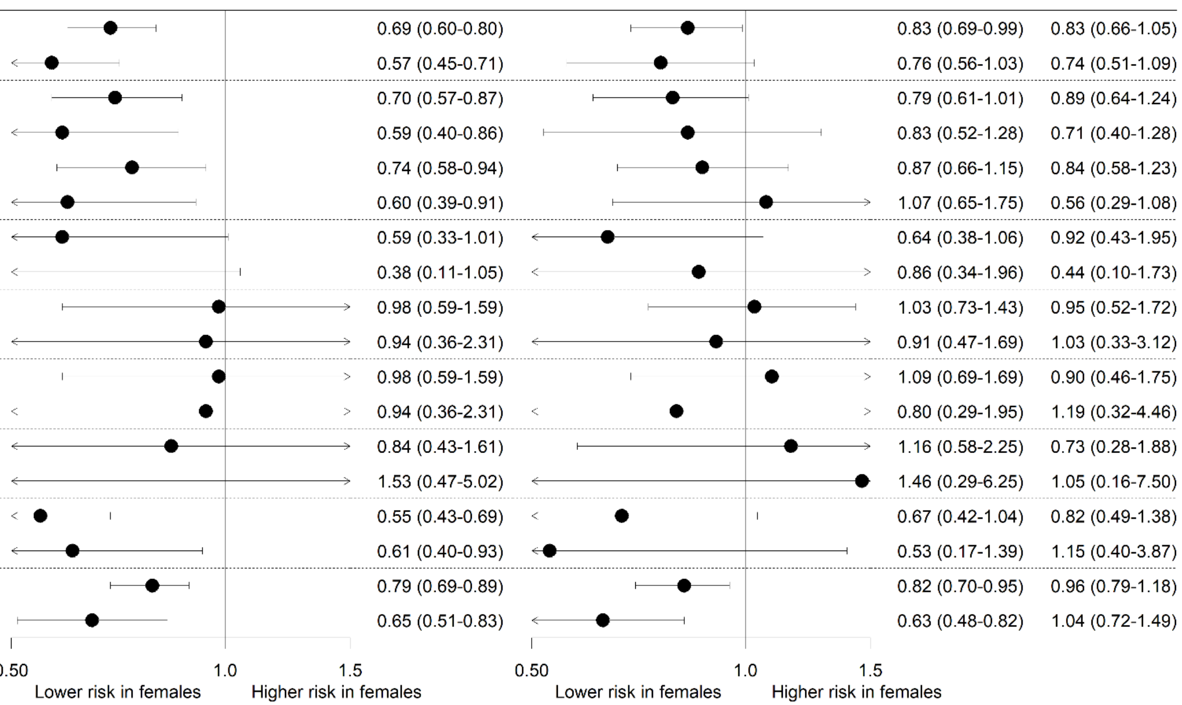

Outcome	Model	N complete	No CVD OR (95% CI)	CVD OR (95% CI)	No CVD:CVD ROR (95% CI)
Any complication	Unadjusted	9998	0.69 (0.60-0.80)	0.83 (0.69-0.99)	0.83 (0.66-1.05)
	Multiple-adjusted	3727	0.57 (0.45-0.71)	0.76 (0.56-1.03)	0.74 (0.51-1.09)
Arrhythmia	Unadjusted	9998	0.70 (0.57-0.87)	0.79 (0.61-1.01)	0.89 (0.64-1.24)
	Multiple-adjusted	2775	0.59 (0.40-0.86)	0.83 (0.52-1.28)	0.71 (0.40-1.28)
SVT	Unadjusted	9990	0.74 (0.58-0.94)	0.87 (0.66-1.15)	0.84 (0.58-1.23)
	Multiple-adjusted	2772	0.60 (0.39-0.91)	1.07 (0.65-1.75)	0.56 (0.29-1.08)
Cardiac ischemia	Unadjusted	9998	0.59 (0.33-1.01)	0.64 (0.38-1.06)	0.92 (0.43-1.95)
	Multiple-adjusted	3563	0.38 (0.11-1.05)	0.86 (0.34-1.96)	0.44 (0.10-1.73)
Heart failure	Unadjusted	9998	0.98 (0.59-1.59)	1.03 (0.73-1.43)	0.95 (0.52-1.72)
	Multiple-adjusted	2838	0.94 (0.36-2.31)	0.91 (0.47-1.69)	1.03 (0.33-3.12)
New-onset heart failure	Unadjusted	9998	0.98 (0.59-1.59)	1.09 (0.69-1.69)	0.90 (0.46-1.75)
	Multiple-adjusted	2838	0.94 (0.36-2.31)	0.80 (0.29-1.95)	1.19 (0.32-4.46)
Stroke	Unadjusted	9998	0.84 (0.43-1.61)	1.16 (0.58-2.25)	0.73 (0.28-1.88)
	Multiple-adjusted	2565	1.53 (0.47-5.02)	1.46 (0.29-6.25)	1.05 (0.16-7.50)
Pulmonary embolism	Unadjusted	9998	0.55 (0.43-0.69)	0.67 (0.42-1.04)	0.82 (0.49-1.38)
	Multiple-adjusted	2565	0.61 (0.40-0.93)	0.53 (0.17-1.39)	1.15 (0.40-3.87)
Death	Unadjusted	10053	0.79 (0.69-0.89)	0.82 (0.70-0.95)	0.96 (0.79-1.18)
	Multiple-adjusted	3743	0.65 (0.51-0.83)	0.63 (0.48-0.82)	1.04 (0.72-1.49)

Supplementary Figure 7 Complete case analyses: Female-to-male odds ratios (ORs, with 95% confidence intervals [CIs]) in the cohorts without and with pre-existing cardiovascular disease (CVD), and the corresponding ratio of OR (with 95% CI). Unadjusted and adjusted estimates are presented. In adjusted analyses, models were adjusted for age, ethnicity, CVD history, medication use and CVD risk factors (hypertension, diabetes, dyslipidemia, peripheral arterial disease and body mass index).

Outcome	Model	N female	Event female	N male	Event male	OR (95% CI)	p-value
Any complication	Unadjusted	4165	519	6208	1038	0.71 (0.64-0.80)	<0.001
	Multiple-adjusted					0.71 (0.63-0.79)	<0.001
Arrhythmia	Unadjusted	4165	202	6208	438	0.67 (0.57-0.80)	<0.001
	Multiple-adjusted					0.65 (0.54-0.77)	<0.001
SVT	Unadjusted	4165	159	6208	325	0.73 (0.60-0.89)	0.001
	Multiple-adjusted					0.69 (0.56-0.84)	<0.001
Cardiac ischemia	Unadjusted	4165	43	6208	114	0.56 (0.39-0.80)	0.001
	Multiple-adjusted					0.56 (0.39-0.80)	0.002
Heart failure	Unadjusted	4165	85	6208	138	0.92 (0.70-1.21)	0.54
	Multiple-adjusted					0.94 (0.71-1.24)	0.65
New-onset heart failure	Unadjusted	4165	63	6208	96	0.98 (0.71-1.35)	0.90
	Multiple-adjusted					0.99 (0.71-1.37)	0.93
Stroke	Unadjusted	4165	31	6208	50	0.93 (0.59-1.46)	0.75
	Multiple-adjusted					0.94 (0.60-1.49)	0.80
Pulmonary embolism	Unadjusted	4165	133	6208	325	0.60 (0.49-0.74)	<0.001
	Multiple-adjusted					0.60 (0.49-0.74)	<0.001
Death	Unadjusted	4165	778	6208	1444	0.76 (0.69-0.83)	<0.001
	Multiple-adjusted					0.66 (0.60-0.74)	<0.001

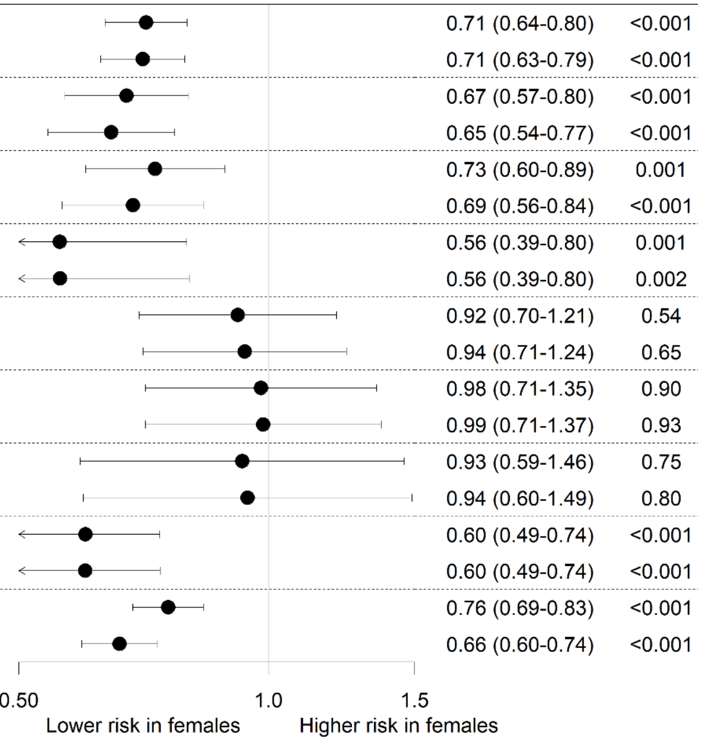

Supplementary Figure 8 Sensitivity analyses with patients from selective recruitment sites excluded: Odds ratios (ORs, with 95% confidence intervals [CIs]) for the association between sex and the outcomes of interest. Unadjusted and adjusted estimates are presented. In adjusted analyses, models were adjusted for age, ethnicity, cardiovascular disease (CVD) history, medication use and CVD risk factors (hypertension, diabetes, dyslipidemia, peripheral arterial disease and body mass index).

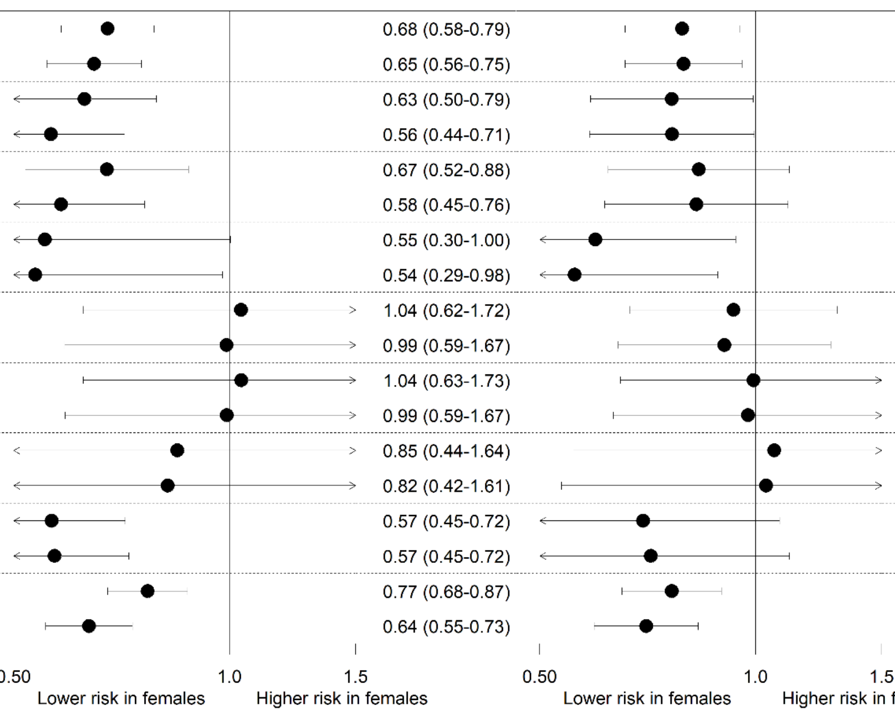

Outcome	Model	No CVD OR (95% CI)	CVD OR (95% CI)	No CVD:CVD ROR (95% CI)
Any complication	Unadjusted	0.68 (0.58-0.79)	0.79 (0.66-0.95)	0.86 (0.67-1.09)
	Multiple-adjusted	0.65 (0.56-0.75)	0.79 (0.66-0.96)	0.82 (0.64-1.04)
Arrhythmia	Unadjusted	0.63 (0.50-0.79)	0.76 (0.59-0.99)	0.82 (0.58-1.17)
	Multiple-adjusted	0.56 (0.44-0.71)	0.76 (0.59-1.00)	0.74 (0.52-1.05)
SVT	Unadjusted	0.67 (0.52-0.88)	0.83 (0.62-1.12)	0.81 (0.55-1.20)
	Multiple-adjusted	0.58 (0.45-0.76)	0.83 (0.62-1.11)	0.70 (0.47-1.05)
Cardiac ischemia	Unadjusted	0.55 (0.30-1.00)	0.60 (0.38-0.94)	0.92 (0.43-1.98)
	Multiple-adjusted	0.54 (0.29-0.98)	0.56 (0.35-0.89)	0.96 (0.44-2.07)
Heart failure	Unadjusted	1.04 (0.62-1.72)	0.93 (0.67-1.30)	1.11 (0.60-2.06)
	Multiple-adjusted	0.99 (0.59-1.67)	0.91 (0.64-1.27)	1.09 (0.59-2.05)
New-onset heart failure	Unadjusted	1.04 (0.63-1.73)	0.99 (0.65-1.52)	1.05 (0.53-2.05)
	Multiple-adjusted	0.99 (0.59-1.67)	0.98 (0.63-1.51)	1.02 (0.51-2.02)
Stroke	Unadjusted	0.85 (0.44-1.64)	1.06 (0.56-2.02)	0.80 (0.31-2.05)
	Multiple-adjusted	0.82 (0.42-1.61)	1.04 (0.54-2.00)	0.79 (0.30-2.09)
Pulmonary embolism	Unadjusted	0.57 (0.45-0.72)	0.70 (0.45-1.08)	0.81 (0.49-1.34)
	Multiple-adjusted	0.57 (0.45-0.72)	0.71 (0.46-1.12)	0.80 (0.48-1.33)
Death	Unadjusted	0.77 (0.68-0.87)	0.76 (0.65-0.90)	1.01 (0.82-1.24)
	Multiple-adjusted	0.64 (0.55-0.73)	0.70 (0.60-0.83)	0.90 (0.73-1.13)

Supplementary Figure 9 Sensitivity analyses with patients from selective recruitment sites excluded: Female-to-male odds ratios (ORs, with 95% confidence intervals [CIs]) in the cohorts without and with pre-existing cardiovascular disease (CVD), and the corresponding ratio of OR (with 95% CI). Unadjusted and adjusted estimates are presented. In adjusted analyses, models were adjusted for age, ethnicity, CVD history, medication use and CVD risk factors (hypertension, diabetes, dyslipidemia, peripheral arterial disease and body mass index).

Lay Summary

Compared to men, women with COVID-19 have a lower risk of severe disease, including respiratory failure, hospitalisation and death. It is less clear whether this is also true for cardiovascular complications associated with COVID-19, for example, irregular heartbeat (arrhythmia), decreased blood flow and oxygen to the heart muscle (cardiac ischemia) and lack of blood flow to the brain (stroke). It is also unclear whether differences in COVID-19 severity between women and men is simply explained by the latter having a higher prevalence of pre-existing cardiovascular disease – a known risk factor for severe COVID-19.

In this study, we analysed data from 11,167 patients who were hospitalised with COVID-19 between March 2020 and May 2021. We compared the risk of a range of cardiovascular complications between women and men and also according to whether patients had pre-existing cardiovascular disease or not.

We found that 13 of every 100 women and 17 of every 100 men developed some form of cardiovascular complication during their hospital admission. This corresponds to a 30% lower risk in women compared to men. Arrhythmia was the most common cardiovascular complication, observed in 5 and 8 of every 100 women and men, respectively. Other cardiovascular complication sub-types, such as cardiac ischemia and pulmonary embolism, were less common overall and were similarly found to occur less frequently in women compared to men. There were also some complications, such as heart failure and stroke, for which women and men had a similar risk.

Importantly, we found that differences between the genders were present irrespective of whether they had pre-existing cardiovascular disease. This suggests that the higher risk in men may not only be explained by their higher prevalence of pre-existing cardiovascular disease. Further research is needed to understand why men are at higher risk of severe COVID-19.

Supplementary References

1. van Buuren S, Groothuis-Oudshoorn K. mice: Multivariate Imputation by Chained Equations in R. *Journal of Statistical Software* 2011;45(3):1 - 67. doi: 10.18637/jss.v045.i03

2. arsenal: An Arsenal of 'R' Functions for Large-Scale Statistical Summaries [program]. 3.6.3 version, 2021.

3. dplyr: A Grammar of Data Manipulation [program]. R package version 1.0.6 version, 2021.

4. forestplot: Advanced Forest Plot Using 'grid' Graphics [program]. R package version 2.0.1 version, 2021.

5. Caforio ALP, Pankuweit S, Arbustini E, et al. Current state of knowledge on aetiology, diagnosis, management, and therapy of myocarditis: a position statement of the European Society of Cardiology Working Group on Myocardial and Pericardial Diseases. *European Heart Journal* 2013;34(33):2636-48. doi: 10.1093/eurheartj/eht210

6. Adler Y, Charron P, Imazio M, et al. 2015 ESC Guidelines for the diagnosis and management of pericardial diseases: The Task Force for the Diagnosis and Management of Pericardial Diseases of the European Society of Cardiology (ESC)Endorsed by: The European Association for Cardio-Thoracic Surgery (EACTS). *European Heart Journal* 2015;36(42):2921-64. doi: 10.1093/eurheartj/ehv318

7. Habib G, Lancellotti P, Antunes MJ, et al. 2015 ESC Guidelines for the management of infective endocarditis: The Task Force for the Management of Infective Endocarditis of the European Society of Cardiology (ESC)Endorsed by: European Association for Cardio-Thoracic Surgery (EACTS), the European Association of Nuclear Medicine (EANM). *European Heart Journal* 2015;36(44):3075-128. doi: 10.1093/eurheartj/ehv319

8. Roffi M, Patrono C, Collet J-P, et al. 2015 ESC Guidelines for the management of acute coronary syndromes in patients presenting without persistent ST-segment elevation: Task Force for the Management of Acute Coronary Syndromes in Patients Presenting without Persistent ST-Segment Elevation of the European Society of Cardiology (ESC). *European Heart Journal* 2016;37(3):267-315. doi: 10.1093/eurheartj/ehv320

9. Buxton AE, Calkins H, Callans DJ, et al. ACC/AHA/HRS 2006 key data elements and definitions for electrophysiological studies and procedures: a report of the American College of Cardiology/American Heart Association Task Force on Clinical Data Standards (ACC/AHA/HRS Writing Committee to Develop Data Standards on Electrophysiology). *J Am Coll Cardiol* 2006;48(11):2360-96. doi: 10.1016/j.jacc.2006.09.020 [published Online First: 2006/12/13]
